# Identification of a novel mitochondria-localized LKB1 variant required for the regulation of the oxidative stress response

**DOI:** 10.1016/j.jbc.2023.104906

**Published:** 2023-06-09

**Authors:** Ivan Tan, Shengli Xu, Jianxin Huo, Yuhan Huang, Hong-Hwa Lim, Kong-Peng Lam

**Affiliations:** 1 Singapore Immunology Network (SIgN), Agency for Science, Technology and Research (A∗STAR), Singapore, Singapore; 2 Department of Physiology, Yong Loo Lin School of Medicine, National University of Singapore, Singapore, Singapore; 3 Institute of Molecular and Cell Biology, Agency for Science, Technology and Research, Singapore, Singapore; 4 Department of Microbiology and Immunology, Yong Loo Lin School of Medicine, National University of Singapore, Singapore, Singapore; 5 School of Biological Sciences, Nanyang Technological University, Singapore, Singapore

**Keywords:** isoforms, intron, exon, cellular localization, mitochondrial respiration, DNA damage

## Abstract

The tumor suppressor Liver Kinase B1 (LKB1) is a multifunctional serine/threonine protein kinase that regulates cell metabolism, polarity, and growth and is associated with Peutz-Jeghers Syndrome and cancer predisposition. The *LKB1* gene comprises 10 exons and 9 introns. Three spliced *LKB1* variants have been documented, and they reside mainly in the cytoplasm, although two possess a nuclear-localization sequence (NLS) and are able to shuttle into the nucleus. Here, we report the identification of a fourth and novel LKB1 isoform that is, interestingly, targeted to the mitochondria. We show that this mitochondria-localized LKB1 (mLKB1) is generated from alternative splicing in the 5′ region of the transcript and translated from an alternative initiation codon encoded by a previously unknown exon 1b (131 bp) hidden within the long intron 1 of *LKB1* gene. We found by replacing the N-terminal NLS of the canonical *LKB1* isoform, the N-terminus of the alternatively spliced *mLKB1* variant encodes a mitochondrial transit peptide that allows it to localize to the mitochondria. We further demonstrate that mLKB1 colocalizes histologically with mitochondria-resident ATP Synthase and NAD-dependent deacetylase sirtuin-3, mitochondrial (SIRT3) and that its expression is rapidly and transiently upregulated by oxidative stress. We conclude that this novel LKB1 isoform, mLKB1, plays a critical role in regulating mitochondrial metabolic activity and oxidative stress response.

Liver Kinase B1 (LKB1), otherwise known as Serine/Threonine Kinase 11 (STK11), is an upstream regulator of AMP-activated protein kinase (AMPK) and 12 other AMPK-related kinases (ARKs) (1, 2, 3). It plays crucial roles in regulating various cellular activities such as metabolism (3, 4, 5), apoptosis (6, 7), cell polarity (8, 9), autophagy (10, 11), cell cycle regulation (12), oxidative stress, and DNA damage response (13, 14, 15, 16). LKB1 is also known to function as a tumor suppressor. Germline mutations in the *LKB1* gene have been linked directly to Peutz-Jeghers syndrome (PJS) (17, 18), a disorder that is characterized by mucosal gastrointestinal polyps development and cancer predisposition (19, 20, 21, 22). Various studies have also uncovered LKB1 mutations in many other types of cancers, including those of the lung, cervix, kidney, and pancreas (22, 23, 24, 25). In particular, mutations resulting in LKB1 inactivation are found in 30% of non-small cell lung cancer (NSCLC, of the Caucasian population) and 20% of cervical carcinoma (26, 27).

The canonical full-length human LKB1 consists of 433 amino acid residues. Structurally, it contains an N-terminal nuclear localization signal (NLS) motif, followed by the kinase catalytic domain and a C-terminal tail with a farnesylation and multiple serine phosphorylation sites (13). While the exact role of the C-terminus is yet to be fully understood, mutations in this region have been shown to be associated with both PJS and sporadic cancers (22, 23, 24, 25, 28). LKB1 forms a heterotrimeric complex with the scaffolding molecule MO25 and the pseudokinase STRADα (29). MO25 interacts with STRADα to enhance its interaction with LKB1, leading to the activation of LKB1 and its nucleocytoplasmic translocation (29, 30). The cytosolic pool of LKB1 is believed to be the active protein that exerts its tumor suppressor functions (31). On the other hand, nuclear LKB1 has been shown to bind other tumor suppressors, such as p53 (16).

To date, three alternatively spliced variants of LKB1 have been identified; the canonical full-length LKB1 that is known as the long-form LKB1_L_ (13), the shorter splice variant LKB1_S_ that contains a shorter C-terminus lacking the farnesylation and a serine phosphorylation sites (32, 33), and the catalytically inactive DN-LKB1 that is devoid of the NLS and has truncation in the kinase domain (34). Both LKB1_L_ and LKB1_S_ are conserved in many aspects, and these include their ability to phosphorylate AMPK and AMPK-related kinases (ARKs), complex with STRADα/MO25, and shuttle between cytosol and nucleus (32). On the other hand, ΔN-LKB1 is found in the cytoplasm but cannot bind STRADα/MO25. However, it can potentiate AMPK activation by LKB1_L_ and has an intrinsic oncogenic property (34).

The gene encoding human *LKB1* is on chromosome 19 and spans a genomic region of more than 50 kb. It consists of 10 exons interspersed by 9 introns (32). The LKB1_L_ isoform is derived from the 10 exons, while LKB1_S_ is generated from alternate usage of the 3′ exon that resulted in a shortened C-terminal lacking Ser428 phosphorylation and Cys430 farnesylation sites (32, 33). The ΔN-LKB1 isoform is produced by alternate splicing in exon 1 that eliminates the start codon leading to the usage of an in-frame translation initiation site in exon 3 (34), resulting in a smaller protein.

Given the gene structure of *LKB1* that spans a vast genomic locus of 50 kb and contains 10 exons and 9 introns of varying lengths with an exceptionally long intron 1, we wonder if other as-yet-to-be uncovered LBK1 isoforms exist. Here, we describe the identification of a novel splice variant of LKB1 that is generated using an alternative initiation start codon encoded by a previously uncharacterized exon in the first intron of the *LKB1* gene. We show that this LKB1 isoform does not harbor an NLS but is instead targeted to the mitochondria. Furthermore, we demonstrate that this mitochondria-localized LKB1 (mLKB1) is catalytically active and is critical in regulating mitochondrial oxidative stress.

## Results

### Identification of a novel exon encoding an alternative translation initiation codon within intron one of *LKB1* gene

The human *LKB1* gene is located on chromosome 19: 1,177,558 to 1,228,431 bp and spans a genomic region of more than 50 kb (Ensembl database). The transcript of the most predominant LKB1 isoform is derived from the 10 exons that are separated by 9 introns. While introns 2 to 7 and intron 9 do not exceed 1 kb in length, introns 1 and 8 are 11,213 bp and 3281 bp long, respectively. Intron 1 of *LKB1* is ten times longer than the median intron length of the human genome, which is known to be around 1.3 kb (35, 36). This unusually long intron 1 of the *LKB1* gene prompted us to examine it closer for hitherto undiscovered exon(s).

**Figure 1:**
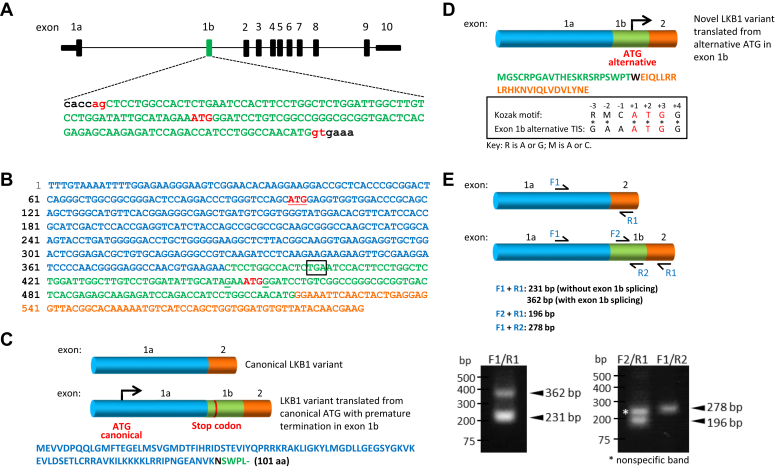
**Identification of a novel exon with an alternative initiation codon embedded in intron one of *LKB1* gene.***A*, schematic diagram of *LKB1* gene depicting the organization of various exons and introns. The original exon one is renamed 1a and the novel exon (in *green*) described in this study is named 1b. Genomic sequence of the putative 131 bp novel exon 1b flanked by short intronic sequences. The putative exon sequence (*green*, *upper case letters*), ATG start codon (*red*, *upper case letters*), intronic sequences (*black*, *lower case letters*), and the conserved 5′-splice donor and 3′-splice acceptor sites (gt-ag, *red lower-case letters*) are shown. *B*, BM819015 EST obtained from the NCBI database with sequences corresponding to *LKB1* exon 1a (*blue*), exon 2 (*orange*), and exon 1b (*green*). The canonical ATG start codon encoded by exon 1a is in *red* and *underlined* and the alternative ATG start codon in exon 1b is in *red*. The premature stop codon encoded in exon 1b is boxed. Conserved Gs at −3 and +4 positions of the Kozak motif of the alternative initiation site are *bolded* and *underlined*. *C*, organization of the spliced transcripts of the canonical *LKB1* and the novel variant with exon 1b (*green*) insertion (sequence from BM819015). The amino acid sequence translated from the canonical ATG start site is shown. Note that translation is terminated prematurely by the stop codon encoded in exon 1b. The amino acid residue N (*black*) resulted from the joining of exons 1a and 1b. *D*, organization of the sequence from BM819015 EST and the amino acid sequence translated from the alternative start codon in exon 1b. The amino acid residue W (*black*) resulted from the joining of exons 1b and 2. Comparison of the translation initiation site (TIS) in exon 1b and the conserved Kozak motif. *E*, verifications of the spliced transcripts corresponding to the canonical and novel *LKB1* variants. Relative annealing positions of primers used for RT-PCR are shown. RT-PCR products amplified from cDNA synthesized from U2OS cells, using primer pairs F1/R1, F2/R1, and F1/R2, for the detection of the transcript of the novel *LKB1* variant with exon 1b insertion. The expected sizes of the PCR products derived from the various primers are shown. A non-specific PCR product is marked by ∗.

Upon inspecting the sequence, we found a possible exon of 131 bp, which we termed exon 1b (Fig. 1*A*, green), located 9064 bp downstream of the original exon 1 (renamed here as exon 1a) and 2018 bp upstream of exon 2. To determine if this potential exon 1b is genuine, we used the BLAST program to search the sequence against the human EST database at NCBI and found two matched EST cDNA sequences (Genebank: BM819015 and AA904306). A closer examination of the two ESTs showed that the exon 1b sequence in AA904306 is flanked by intronic sequences (data not shown), suggesting that this EST was likely reverse-transcribed from a nascent RNA. Interestingly, the exon 1b sequence (Fig. 1*B*, green) in BM819015 is inserted between those of exons 1a (blue) and 2 (orange) of *LKB1* (Fig. 1, *B* and *C*). These findings suggest that exon 1b could undergo alternative splicing and form part of a previously unappreciated mRNA variant of *LKB1*. Further analysis of BM819015 reveals that mRNA translation initiated from the canonical ATG (Fig. 1*B*, in red and underlined) encoded in exon 1a would lead to a premature termination codon (Fig. 1*B*, boxed) located within exon 1b and resulting in a truncated polypeptide of only 101 amino acid residues (Fig. 1*C*).

Interestingly, we noticed the presence of a putative translation initiation site within exon 1b (Fig. 1, *A*, *B* and *D*), which shows significant homology to the Kozak consensus sequence (37), including the two conserved G residues at −3 and +4 positions on either side of the adenosine residue (numbered +1) of the putative ATG start site (Fig. 1*D*). Importantly, the reading frame arising from this alternative ATG start codon in exon 1b is contiguous with the reading frame in exon 2 (Fig. 1, *B* and *D*), thus bolstering support for its possible use as an alternative initiation site. Hence, our analysis so far suggests the existence of a putative exon (exon 1b), which contains a putative alternative translation initiation site within intron 1 of the *LKB1* gene.

### Detection of a smaller LKB1 protein variant generated from the alternative translation initiation site in exon 1b

Next, we checked if exon 1b could exist as part of *LKB1* transcripts by performing reverse transcription-PCR (RT-PCR) using cDNA synthesized from human osteosarcoma U2OS cells. As shown in Figure 1*E*, PCR products obtained from primer pair (F1 and R1) specific for exons 1a and 2 of *LKB1* gene yielded two bands: a major band with the expected size of 231 bp and a minor band of 362 bp. DNA sequencing of these two bands indicated that the major 231 bp band was derived from the canonical *LKB1* transcript while the minor 362 bp band was the product of an *LKB1* mRNA variant harboring the 131 bp sequence from exon 1b inserted between exons 1a and 2 (data not shown). The presence of exon 1b in *LKB1* transcripts was further confirmed by RT-PCR and DNA sequencing using primer pairs specific for exons 1a and 1b (F1 and R2) and for exons 1b and 2 (F2 and R1) as shown in Figure 1*E*. Thus, our data showed that exon 1b could indeed form part of mature *LKB1* mRNA transcripts.

**Figure 2:**
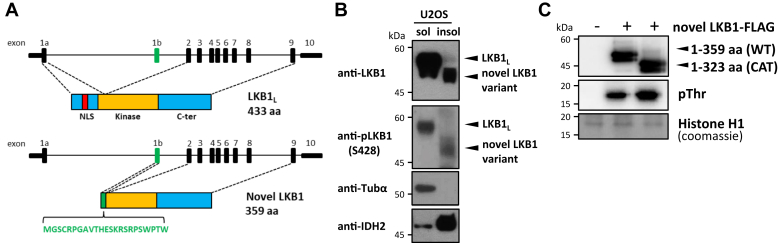
**Generation of a novel LKB1 isoform from alternative translation start site encoded in exon 1b.***A*, schematic diagram showing the genomic and protein domain structures of human LKB1. Corresponding exon and protein subdomains are connected by *dotted lines*. Translation initiation from the use of the canonical ATG start codon encoded in exon 1a produces the full-length LKB1_L_ protein (433 amino acids). Translation initiation from the alternative ATG start codon in exon 1b produces the novel LKB1 variant (359 amino acids). The amino acid sequence encoded by exon 1b is shown. *B*, Western blot analysis of LKB1 protein. Triton X-100-soluble (20 mg) and -insoluble (50 mg) fractions of U2OS cells were resolved on SDS-PAGE and probed with two different LKB1 antibodies as indicated. The anti-tubulin-a and anti-IDH2 blots were included as controls for the fractionation of cellular lysates. *C*, *in vitro* kinase assay of overexpressed novel LKB1 variant. Full-length (WT) and kinase domain (CAT) of novel LKB1 variant were immunoprecipitated with FLAG antibody-coated agarose and incubated with Histone H1 as substrate. Proteins were resolved on SDS-PAGE and the phosphorylation of histone H1 was examined using an anti-phospho-Thr antibody. aa, amino acid; C-ter, C-terminal; NLS, nuclear localization signal.

As a result of the exclusion of the entire coding sequence in exon 1a, the predicted size of the novel LKB1 variant translated from the alternative initiation start site in exon 1b is expected to be smaller at 359 amino acids compared to LKB1_L_ at 433 amino acids in size. Moreover, it will lack the NLS as well as part of the kinase domain N-lobe (Fig. 2*A*). To ascertain the functionality of this alternative initiation site in exon 1b and to determine if the novel *LKB1* variant is translated and can exist, we incubated U2OS cells with Triton X-100 (TX) and fractionated the cell lysates into TX-soluble and insoluble fractions before gel electrophoresis and western blotting. We subsequently probed the cell lysates with two different antibodies: an anti-LKB1 antibody (clone D60C5) to detect all forms of LKB1 and an anti-phospho-LKB1(Ser428) antibody to detect phosphorylated LKB1. These two antibodies were used in this study to independently verify the existence of the novel LKB1 variant, as we do not yet possess an antibody specific to this putative isoform. Moreover, we have mapped the antigen-binding sites of these two antibodies to the C-terminus of LKB1 (Fig. S1), which is unaffected by the splicing event; hence, these two antibodies could potentially identify the novel LKB1 isoforms based on their sizes. As shown in Figure 2*B*, the two antibodies consistently detected LKB1_L_ (∼57 kDa) in the TX-soluble fraction of U2OS cells (Fig. 2*B*, lane 1), consistent with previous reports (6, 12). In addition, they also detected protein bands corresponding to the smaller novel LKB1 variant (∼48 kDa), interestingly, in the TX-insoluble fraction of the cells (lane 2). These data indicated that the alternative translation initiation site encoded in exon 1b is indeed functional and that a novel smaller variant LKB1 can be generated and, surprisingly, localized to a different cellular compartment compared with LKB1_L_.

As a result of the skipping of exon 1a, the novel LKB1 variant does not contain the conserved G-loop and the Lys residue of β-strand 3 of the kinase N-lobe. We, therefore, examined if the new variant possesses kinase activity by testing it on the Histone H1 protein. As shown in Figure 2*C*, the LKB1 variant (1–359 aa) and its kinase domain (1–323 aa), immunoprecipitated from overexpressing cells, were able to phosphorylate Histone H1 *in vitro*, suggesting that this LKB1 isoform is active. This is perhaps not unusual as atypical protein kinases such as mTOR and WNK are also known to be catalytically active while lacking either the conserved G-loop or the β-strand three lysine (38, 39, 40).

### Localization of the novel LKB1 variant to the mitochondria

**Figure 3:**
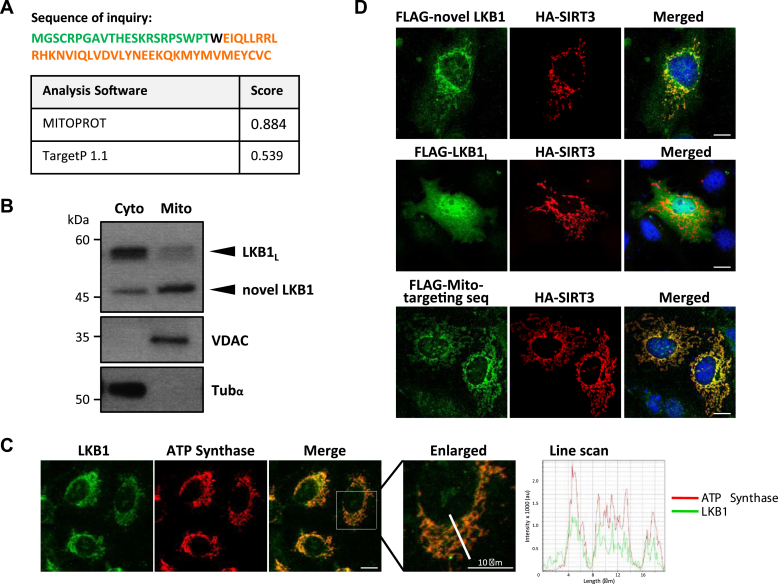
**Mitochondria targeting of novel LKB1 variant.***A*, prediction of a mitochondrial targeting sequence in the N-terminus of novel LKB1 variant using online MITOPROT and TargetP 1.1 analysis software. The sequence corresponding to N-terminal 60 amino acid residues of the novel LKB1 variant was used in the analyses. Amino acids encoded by exon 1a (*green*), exon 1b (*orange*) and residue resulting from merging of exons 1a and b (*black*) are shown. *B*, enrichment of the smaller novel LKB1 variant in the mitochondrial fraction. Cytosolic and mitochondria-enriched fractions of U2OS cells were resolved on SDS-PAGE and probed with an anti-LKB1 antibody. The anti-VDAC and anti-tubulin-α blots were included as controls for the fractionation of cell lysates. *C*, colocalization of novel LKB1 variant with mitochondria-resident ATP Synthase. U2OS cells were permeabilized with 0.1% Triton X-100 for 10 to 15 s to deplete cytosolic LKB1F_L_, fixed with cold methanol at −20 °C and immune-stained with anti-LKB1 and anti-ATP Synthase antibodies overnight. Line scan analysis of boxed cell is shown. Scale bar: 10 μm. *D*, localization of over-expressed LKB1 variant to mitochondria. Cos-7 cells were co-transfected with HA-tagged SIRT3 and FLAG-tagged mLKB1 variant or FLAG-tagged LKB1_L_ or FLAG-tagged N-terminal putative mitochondria transit peptide. Thereafter, cells were stained with DAPI (*blue*) and fluorochrome-conjugated anti-HA (*red*) and anti-FLAG (*green*) antibodies to visualize the overexpressed proteins and peptides. Scale bars: 10 μm. Images were acquired using Olympus Fluoview1000 confocal microscope.

Given the unexpected finding that this novel LKB1 variant resides in the TX-insoluble fraction of the cell and our earlier analysis that the usage of the alternative translation initiation site in exon 1b would eliminate the NLS, we proceeded to examine in detail its N-terminal amino acid sequence for any organelle-specific targeting signal. Predictions from online analysis tools such as MITOPROT and TargetP-1.1 (Fig. 3*A*) suggested that the N-terminal amino acid sequence of this novel LKB1 variant might encode a mitochondrial transit peptide that facilitates the localization of proteins to the mitochondria (41). To address this possibility, we first determined if this novel LBK1 variant can be detected endogenously in the mitochondria using Western blot analysis. Subcellular fractions corresponding to cytosolic and mitochondrial fractions of U2OS cells were prepared and probed for the presence of LKB1_L_ and this novel LKB1 variant. As shown in Figure 3*B*, while most of LKB1_L_ was detected in the cytosolic fraction marked by tubulin-α, the smaller novel LKB1 variant was found enriched in the mitochondrial fraction marked by the voltage-dependent anion channel (VDAC) protein.

To further substantiate this finding, we went on to determine if endogenous LKB1 can be detected histologically in the mitochondria aside from our fractionation and western blotting study in Figure 3*B*. We first briefly permeabilized U2OS cells with Triton X-100 to deplete cytosolic LKB1 before fixation and staining with antibodies. This step was undertaken as we do not have a specific antibody to this LKB1 variant and would rely on using the anti-LKB1 antibody to detect the presence of the LKB1 variant in the mitochondria. As shown in Figure 3*C*, immunostaining of Triton X-100-permeabilized U2OS cells with an anti-LKB1 antibody showed a strong LKB1 staining in the mitochondria that colocalized well with the staining of the mitochondrial resident protein ATP Synthase, further providing evidence for the mitochondrial localization of this endogenous LKB1 variant. In addition, the novel LKB1 variant overexpressed from cloned cDNA behaved similarly to the endogenous protein regarding both Triton X-100 solubility and migration on SDS-PAGE (Fig. S2).

Finally, to definitively corroborate the localization of this novel LKB1 variant to the mitochondria, FLAG-tagged versions of this novel LKB1 variant and its N-terminal sequence alone were separately co-expressed with the mitochondria-localized HA-tagged SIRT3 in Cos7 cells (42) and examined by fluorescence microscopy. As shown in Figure 3*D*, both the novel LKB1 variant and its N-terminal sequence colocalized strongly with SIRT3, indicating that they were primarily localized in the mitochondria. In contrast, FLAG-tagged LKB1_L_ was mainly detected in the cytoplasm and the nucleus. Taken together, our results show that this smaller novel LKB1 variant translated from the alternate translation initiation start site in exon 1b could be targeted to the mitochondria through the mitochondrial transit peptide motif present at the N-terminus. Hence, we decide to denote this novel mitochondria-localized LKB1 variant as mLKB1.

### Expression of mLKB1 is upregulated by oxidative stress

LKB1 regulates oxidative stress in cells and protects cells from oxidative damage (10, 15). Mitochondria are the known major intracellular sites where oxidative stress is generated due to the activity of the electron transport chain (43, 44). Therefore, we hypothesized that the mitochondria-targeted mLKB1, whose expression could be readily detected in multiple cell lines of different tissue origins (Fig. S3), could play a role in the regulation of oxidative stress generated in the mitochondria.

**Figure 4:**
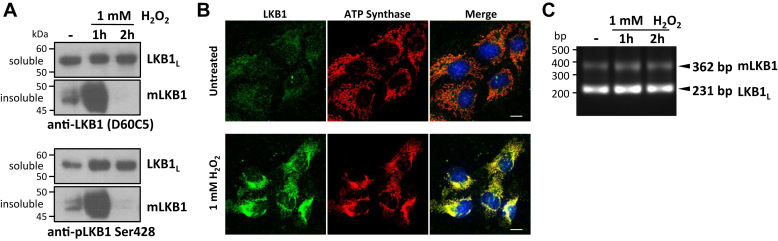
**Expression of mLKB1 is regulated by oxidative stress.***A*, upregulation of mLKB1 protein expression by H_2_O_2_ treatment. U2OS cells were treated with 1 mM H_2_O_2_ for 1 and 2 h, fractionated into Triton X-100-soluble and -insoluble fractions, and probed with the two separate LKB1 antibodies as indicated. *B*, increased mLKB1 in mitochondria upon H_2_O_2_ stimulation. U2OS cells were treated with 1 mM H_2_O_2_ for 1 h, then permeabilized by Triton-X 100-containg PEM buffer and immunostained with antibodies against LKB1 and ATP Synthase overnight. Images were acquired using Olympus Fluoview1000 confocal microscope. Scale bars: 10 μm. *C*, examination of mLKB1 transcripts in H_2_O_2_ treated U2OS cells. Total RNAs obtained from U2OS cells treated with H_2_O_2_ for 1 and 2 h were subjected to RT-PCR using the F1/R1 primer pair in Figure 1*E* to amplify LKB1_L_ and mLKB1 transcripts.

To test this hypothesis, we first examined the effect of hydrogen peroxide (H_2_O_2_) treatment on mLKB1 expression in U2OS cells. Treatment with H_2_O_2_ is a widely used experimental protocol to induce oxidative stress in cells (45). As shown in Figure 4*A*, we found the protein expression level of endogenous mLKB1 to be dynamically regulated by H_2_O_2_ treatment. As detected by the two independent anti-LKB1 antibodies that we had used earlier (Fig. 2*B*), we found mLKB1 expression level to be markedly increased after 1 h of H_2_O_2_ treatment, and it was rapidly down-regulated thereafter (Fig. 4*A*). Consistent with the Western blot data, a similar increase in the expression level of mitochondria-localized LKB1 was also detected, as revealed by the more intense immunofluorescence staining of LKB1 (in green) and its colocalization staining with the mitochondria-resident ATP Synthase (in red), upon H_2_O_2_ exposure (Fig. 4*B*). The increase in mLKB1 expression could be post-translationally regulated as the level of mLKB1 transcript was unaffected by H_2_O_2_ treatment (Fig. 4*C*). Together, these findings show that the expression of the mLKB1 is regulated by H_2_O_2_, supporting the possible involvement of mLKB1 in the regulation of mitochondrial oxidative stress.

### mLKB1 regulates the mitochondrial metabolic activity and oxidative stress

**Figure 5:**
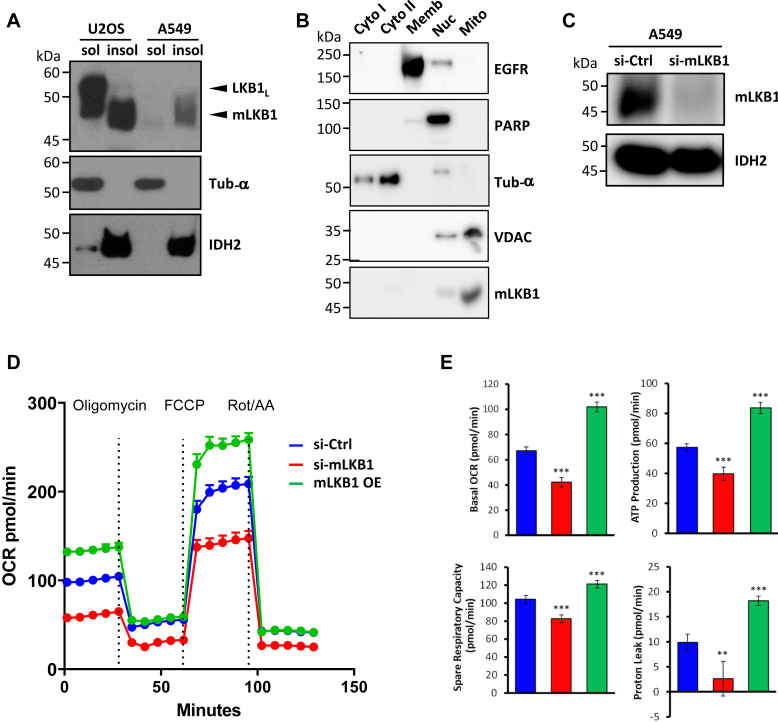
**mLKB1 is required for the regulation of mitochondrial metabolic activity.***A*, A549 cells possess only mLKB1 isoform. Triton X-100-soluble and -insoluble fractions of U2OS and A549 cells were resolved on SDS-PAGE and probed with an anti-LKB1 antibody. The anti-tubulin-a or anti-IDH2 blots were included as controls for the fractionation of cell lysates. Protein extracts loaded were 20 mg for lanes one and 3 and 50 mg for lanes two and 4. *B*, detection of mLKB1 in a mitochondria-enriched fraction of A549 cells. Various subcellular fractions obtained from A549 cells were resolved on SDS-PAGE and probed with anti-LKB1 antibody and other various antibodies against specific organelle to mark subcellular fractions: anti-EGFR for membrane; anti-PARP for nucleus; anti-Tub-a for cytosol and anti-VDAC for the mitochondrial fraction. Cyto I, 12 kg supernatant; Cyo II, 450 kg supernatant. *C*, Western blot analysis of mLKB1 knockdown in A549 cells. Cells were transfected with scrambled siRNA as control (si-Ctrl) or mLKB1-specific siRNA (si-mLKB1). Triton X-100-insoluble fractions were prepared and probed for mLKB1 expression with an anti-LKB1 antibody. The anti-IDH2 blot was included as a control. *D*, real-time analysis of the Oxygen Consumption Rate (OCR) of A549 cells transfected with scrambled siRNA (si-Ctrl, *blue*), mLKB1-specific siRNA (si-mLKB1, *red*), or mLKB1 expression vector (mLKB1 OE, *green*). OCR was measured with the consecutive addition of oligomycin (1 mM), mitochondrial uncoupler FCCP (500 nM), and inhibitors of the mitochondrial electron-transport complex I and III, rotenone and antimycin (R&A, 500 nM each). *E*, quantifications of basal mitochondrial respiration, ATP production, spare respiratory capacity, and proton leak. Data are represented as Mean ± SD (n = 6), and samples were compared using independent Student's *t*-tests; ∗∗*p* < 0.01; ∗∗∗*p* < 0.001.

**Figure 6:**
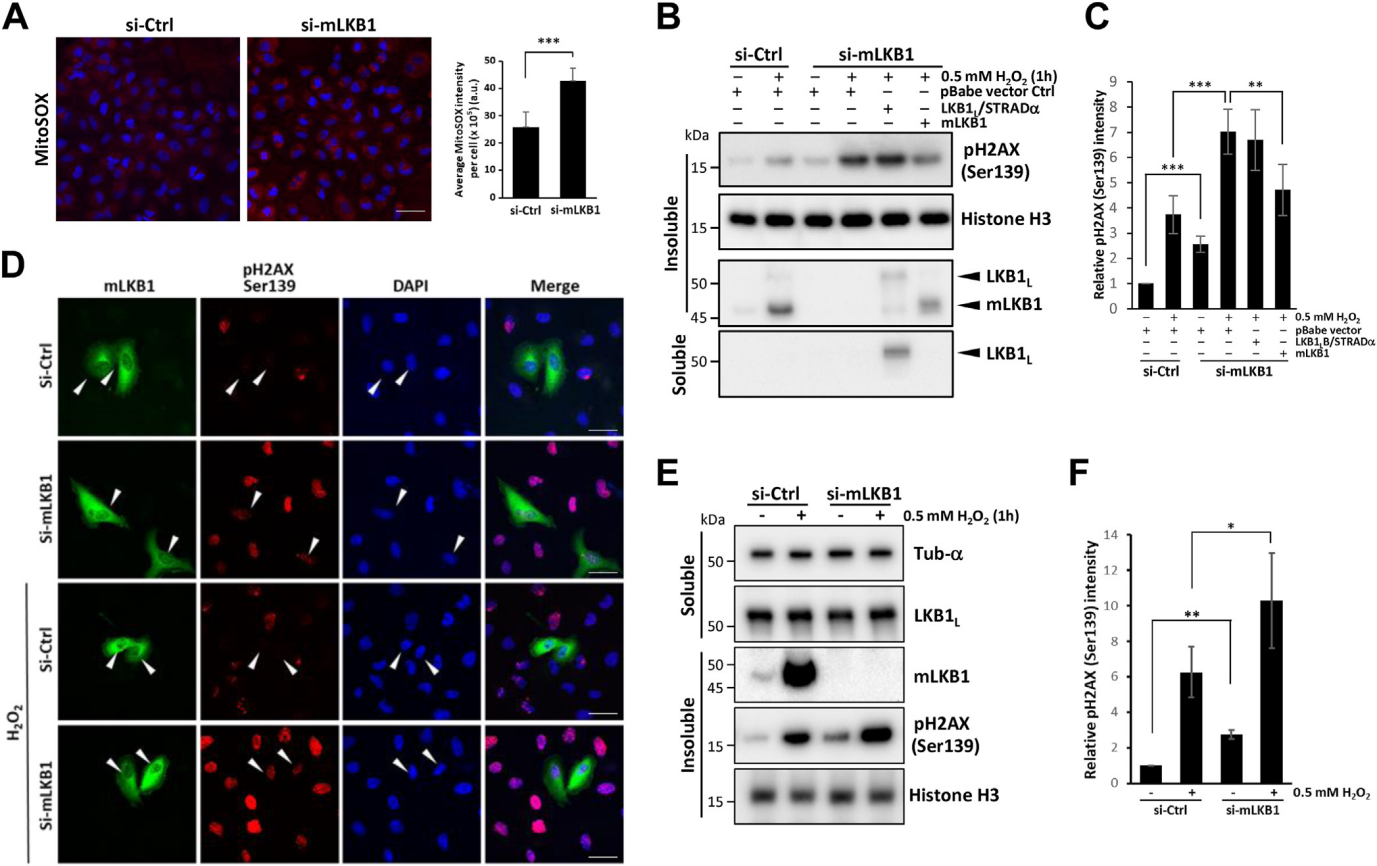
**mLKB1 regulates mitochondrial oxidative stress.***A*, increased oxidative stress in mLKB1-knockdown A549 cells. Visualization (*left panel*) and quantification (*right panel*) of mitochondrial oxidative stress in control and mLKB1 knockdown cells using MitoSOX staining. Scale bar: 50 μm. *B*, enhanced DNA damage in mLKB1-knockdown A549 cells. Western blot analysis of the levels of H_2_O_2_-induced DNA damage in A549 cells transfected with si-Ctrl or si-mLKB1, co-transfected (24 h later) with either an empty pBabe vector, FLAG-LKB1_L_/HA-STRADα constructs or an untagged siRNA-resistant mLKB1 construct (rescue) as indicated, using anti-pH2AX Ser139 antibody. The expression of endogenous mLKB1, reconstituted mLKB1, and LKB1_L_ were detected by an anti-LKB1 antibody. The anti-Histone H3 blot was included as a loading control. *C*, quantification and statistical analysis of data in (*B*). Data are represented as Mean ± SD (n = 3), and samples were compared using independent Student's *t*-tests; ∗∗∗*p* < 0.001; ∗∗*p* < 0.01. *D*, visualization of DNA damage in A549 cells depleted of, or over-expressing mLKB1 in response to H_2_O_2_. A549 cells transfected with si-Ctrl or si-mLKB1 were co-transfected with untagged siRNA-resistant mLKB1 construct, followed by 0.5 mM H_2_O_2_ treatment for 1 h and stained with DAPI, anti-LKB1, and anti-pH2AX Ser139 antibodies. *Arrows* indicate transfected cells overexpressing mLKB1. Scale bars: 25 μm. Images were acquired using Olympus Fluoview1000 confocal microscope. *E*, enhanced DNA damage in mLKB1-knockdown U2OS cells. Western blot analysis of the levels of H_2_O_2_-induced DNA damage in U2OS cells transfected with si-Ctrl or si-mLKB1 using anti-pH2AX Ser139 antibody. Triton X-100 soluble and insoluble fractions were analyzed by separate antibodies as indicated. *F*, quantification and statistical analysis of data in (*E*). Data are represented as Mean ± SD (n = 3), and samples were compared using independent Student's *t* tests; ∗*p* < 0.05; ∗∗*p* < 0.01.

To ascertain the importance of mLKB1 in regulating mitochondrial oxidative stress, we examined human lung carcinoma epithelial A549 cells, which were reported not expressing LKB1_L_ due to the presence of a homozygous nonsense mutation in exon 1a of the *LKB1* gene (46, 47). As the alternative translation start site encoded by exon 1b is still intact, we predicted that the expression of mLKB1 would not be affected in A549 cells. Indeed, as shown in Figure 5*A*, the expression of mLKB1, but not that of LKB1_L_, was detectable in A549 cells. In contrast, U2OS cells possess both LKB1_L_ and mLKB1. And consistent with the finding in U2OS cells (Fig. 3*B*), mLKB1 is also highly enriched in the mitochondria but not in other subcellular fractions of A549 cells (Fig. 5*B*). Furthermore, transfection of mLKB1-specific siRNA led to the depletion of mLKB1 in A549 and U2OS cells without affecting the expression of the conventional LKB1_L_ in U2OS cells (Figs. 5*C* and 6*E*) confirming the existence of the mLKB1 variant in these cells and providing us with a means to study the effect of mLKB1 knockdown in the regulation of mitochondrial metabolic activity and oxidative stress.

We proceeded to study the mitochondrial functions of mLKB1 in A549 cells. First, we examined the oxygen consumption rate (OCR) of A549 cells either depleted of or overexpressing mLKB1, using a Seahorse extracellular flux analyzer. As shown in Figure 5, *D* and *E*, while the basal mitochondrial OCR was down-regulated in mLKB1-knockdown cells compared to control cells transfected with scrambled siRNA, mLKB1-overexpressing cells exhibited increased basal mitochondrial respiration. Likewise, parameters such as ATP production, proton leak that regulates mitochondrial reactive oxygen species (ROS) generation, and spare respiratory capacity, which measures the ability of a cell to respond to increasing energy demands, also manifested similar trends. The opposite effects seen with mLKB1 depletion and overexpression strongly suggest that mLKB1 is involved in regulating mitochondrial metabolic activity.

We next examined the level of mitochondrial stress in mLKB1-sufficient and depleted A549 cells using MitoSOX staining to detect and quantify the levels of ROS generated. As shown in Figure 6*A*, mLKB1-depleted A549 cells were stained more intensely and showed increased production of mitochondrial ROS compared to control cells transfected with scrambled siRNA. An increase in ROS may lead to more DNA damage in cells (48), which can be detected by histone H2AX Ser139 phosphorylation using a specific antibody. Indeed, a more significant amount of histone H2AX Ser139 phosphorylation, indicative of higher levels of DNA damage, was found in mLKB1-depleted A549 cells compared to control cells (Fig. 6*B* lanes one and 3). Furthermore, treatment with H_2_O_2_ also led to significantly higher H2AX Ser139 phosphorylation in mLKB1-depleted cells compared to untreated cells and similarly treated mLKB1-sufficient cells (Fig. 6*B* lanes 2, 3, and 4). These results suggest that mLKB1 prevents mitochondrial ROS production and DNA damage.

To further substantiate the critical role of mLKB1 in inhibiting DNA damage, we reintroduced mLKB1 into A549 cells with depleted mLKB1. We expressed either mLKB1 or the LKB1_L_ variant using a promoterless pBABE vector, which allows for gene expression at lower levels than other vectors (49). This approach maintained the mLKB1 protein at or close to its endogenous level. We observed that the promoterless pBabe vector led to mLKB1 protein expression slightly below its endogenous level, rather than causing an excessive overexpression of mLKB1 (Fig. 6*B* lanes two and 6). Notably, the reconstitution of mLKB1 reduced H_2_O_2_-induced DNA damage in A549 cells with depleted mLKB1 (Fig. 6, *B* and *C*). In contrast, reconstitution of the LKB1_L_/STRADα complex in mLKB1-depleted cells did not significantly reduce H_2_O_2_-induced H2AX phosphorylation. These findings corroborate that mLKB1 is responsible for preventing H_2_O_2_-induced DNA damage, and the conventional LKB_L_ cannot replace its function.

The increased DNA damage in mLKB1-depleted A549 cells with or without further enhancement by H_2_O_2_ treatment and the 'rescue' effect of re-expressing mLKB1 can also be visualized *via* confocal microscopy staining of H2AX Ser139 phosphorylation (Fig. 6*D*). We also performed siRNA-mediated mLKB1 knockdown in U2OS cells that possess both mLKB1 and LKB1_L_. Consistent with the above results, the depletion of mLKB1 in U2OS cells also led to increased H2AX Ser139 phosphorylation in these cells which was further enhanced upon H_2_O_2_ treatment (Fig. 6, *E* and *F*), although these cells have an intact expression of LKB1_L_. Taken together, our findings indicate that mLKB1 is involved in the regulation of mitochondrial oxidative phosphorylation and stress which is crucial for cell survival.

## Discussion

It has been estimated that more than 90% of human genes with multiple exons have alternative splice forms (50), and together with post-translational modifications of various types, they represent the major mechanisms by which a higher order of protein diversity is generated. Here we describe the identification of a novel LKB1 variant generated from the use of an alternative translation initiation codon encoded by a previously unknown novel exon (exon 1b) located in the first intron of the *LKB1* gene. Our results show that this novel LKB1 variant contains a mitochondria-targeting peptide and is involved in the regulation of mitochondrial oxidative stress. We named this novel LKB1 isoform the mitochondria-localized LKB1 (mLKB1) variant.

Previous studies have shown that three variants of LKB1 exist, including the predominant full-length LKB1/LKB1_L_ (13), LKB1_S_ with an alternatively spliced C-terminus (32), and ΔN-LKB1 that has an N-terminal truncation (34). While ΔN-LKB1 is generated through the use of an alternative in-frame translation initiation codon located in exon 3, LKB1_S_ and the mLKB1 described here were identified from alternative exons hidden in the long intron 8 (32) and intron 1, respectively. This is consistent with the idea that major-form exons are more likely to be contained in short introns (<400 bp), while novel or minor exons are contained in long introns (>1000 bp) (51). It should also be mentioned that the splicing of the 131 bp exon 1b described here has been detected in two previous studies (52, 53). However, the insertion was regarded as the product of an aberrant splicing event under nonsense-mediated mRNA decay regulation and was not investigated further. In particular, there was no documentation of these "aberrant transcripts" being translated into LKB1 isoforms.

The cellular localization of LKB1_L_ and LKB1_S_ and their regulation by the STRADα/MO25 complex are conserved (32), and so are their phosphorylation and regulation of downstream target AMPK and other ARKs (32). As such, the difference in their physiological roles has been suggested to reside mainly in their differential tissue distribution, with LKB1_L_ ubiquitously expressed and LKB1_S_ expressed predominantly in the testis. Consistent with its expression pattern, the deletion of LKB1_S_ leads to defective spermatogenesis and infertility in male mice (33, 54). On the other hand, the recently identified ΔN-LKB1 isoform could mediate the phosphorylation of AMPK but not ARKs, and it possesses oncogenic properties (34). And unlike LKB1_L_ and LKB1_S_, which could shuttle from cytoplasm to nucleus as they possess NLS, ΔN-LKB1 resides mainly in the cytosol. These findings suggest that different LKB1 isoforms could have different cellular localizations and functions.

The novel mLKB1 variant we identified in this study provides yet an additional mode of LKB1 regulation and function. We show here for the first time that LKB1 could be targeted to the mitochondria by virtue of the mitochondrial transit peptide encoded by a sequence and translated through an alternative start site found in the novel exon 1b. Consistent with its mitochondrial localization, we show that mLKB1 is vital for overall mitochondrial respiration. Its depletion resulted in compromised respiratory functions and increased mitochondrial stress, as evidenced by decreased basal mitochondrial respiration, ATP-linked respiration/ATP production, and spare respiratory capacity. The drop in maximal respiratory capacity is consistent with the decrease in ATP production. Furthermore, our findings also suggest that mLKB1 plays a critical role in regulating oxidative stress as cells depleted of mLKB1 are more vulnerable to ROS exposure, as evidenced by higher levels of DNA damage in these cells upon H_2_O_2_ treatment.

The different LKB1 isoforms, identified previously and in this study, are likely to engage different downstream targets and activate distinct signaling pathways. Our preliminary analyses show that mLKB1 is catalytically active (Fig. 2*C*), but unlike LKB1_L_, it could not phosphorylate AMPK (Fig. S4). Hence, it will be interesting to elucidate the targets of mLKB1 in the mitochondria as they are likely to play essential roles in cellular energy homeostasis. This effort is currently underway. In addition, as LKB1-deleted tumors have recently been shown to be sensitive to increased oxidative stress or ROS levels (55), it would be necessary to understand better the mechanism underlying the regulation of oxidative stress by mLKB1 to fully exploit the vulnerability of LKB-null cells in cancer treatment.

## Experimental procedures

### cDNA constructs, siRNA, reagents, and antibodies

Wild-type LKB1_L_ and SIRT3 constructs were obtained from Addgene. The cDNA of the novel mLKB1 variant was obtained by RT-PCR using cDNA derived from U2OS cells with primer pair: 5′-CTCACGAGAGCAAGAGATCCAGACCATC-3′ and 5′-CTGTCCATTGTGACTGGCCTCCTC-3'. PCR reactions were carried out using Neo KOD enzyme obtained from TOYOBO with an initial denaturing step of 94 °C for 2 min, followed by 40 cycles of 10 s denaturation at 98 °C, 30 s annealing at 57 °C and 1 min extension at 68 °C. For the amplification/detection of LKB1 exon 1b, the following primers were used: F1, 5′-CCAAGCTCATCGGCAAGTACCTGATG-3′; F2, 5′-CTCTGAATCCACTTCCTGGCTCTGGATTG-3′; R1, 5′-CGTTGTATAACACATCCACCAGCTGGATGAC-3′; R2, 5′-GGTCTGGATCTCTTGCTCTCGTGAGTC-3′. The PCR conditions used were as described above. si-mLKB1 sequence: 5′-AAGAGAUCCAGACCAUCCUGG-3′. Antibodies against LKB1 (D60C5), pLKB1-Ser428 (C67A3), IDH2, and pH2AX-Ser139 were obtained from Cell Signaling Technologies. Antibodies against ATP Synthase beta and VDAC were purchased from Thermo Fisher Scientific and Santa Cruz Biotechnology, respectively. The anti-FLAG antibody and FLAG antibody-conjugated agarose beads were obtained from Sigma-Aldrich. The MitoSOXRed kit was purchased from Thermo Fisher Scientific.

### Cell culture, MitoSOX treatment, and immunofluorescence staining

U2OS, A549, and Cos7 cells were maintained at 37 °C with 5% CO_2_ in Dulbecco's modified Eagle's medium supplemented with 10% fetal bovine serum. Transfection of Cos7 cells with plasmid DNA was performed using Lipofectamine 3000 (Thermo Fisher Scientific) according to the manufacturer's instructions. MitoSOX treatment was performed according to the manufacturer's instructions. Briefly, cells were treated with 5 μM MitoSOX for 10 min, followed by two washes, and finally fixed with 4% paraformaldehyde. For immunofluorescence staining, cells were generally seeded overnight on acid-washed coverslips before experiments were conducted. For the immunostaining of endogenous mitochondrial mLKB1, U2OS cells were permeabilized for 10 s with cytoskeleton stabilizing buffer (100 mM PIPES-NaOH pH 6.9, 1 mM MgCl_2_, 1 mM EGTA, and 0.1% Triton) to deplete cytoplasmic LKB1_L_ followed by fixation in neat cold methanol for 10 min before incubating with anti-LKB1 (D60C5) antibody. Cells expressing transfected constructs were identified by immunostaining the N-terminal tag fused to the constructs using the appropriate primary and secondary antibodies. Alexa 488-conjugated secondary antibodies against mouse and rabbit immunoglobulins were obtained from Thermo Fisher Scientific. Cy3-conjugated anti-mouse and anti-rabbit secondary antibodies were obtained from the Jackson ImmunoResearch Laboratory. Immunofluorescence images were captured using Olympus Fluoview 1000 confocal microscope. Image analyses and measurements were performed using Olympus Fluoview 1000 software and ImageJ.

### Western blotting

Western blotting was performed as described previously (56). Protein samples were resolved by SDS-PAGE, transferred onto the PVDF membrane, and probed overnight using various antibodies as indicated. Triton X-100 soluble and insoluble cell extracts were prepared as follows, cells were first lysed for 5 min in a buffer containing 25 mM Hepes (pH7.3), 0.15 M NaCl, 1.5 mM MgCl_2_, 0.2 mM EDTA, 20 mM β-glycerol phosphate, 1 mM sodium orthovanadate, 0.3% Triton X-100, 5% glycerol and supplemented with protease inhibitor cocktail and calyculin A and followed by centrifugation at 13,000 rpm for 10 min at 4 °C. The resultant supernatant was collected as a TX-soluble fraction. Pellets were resuspended in 1.5X SDS-PAGE sample loading buffer, heated at 95 °C for 10 min, and centrifuged at maximum speed for 10 min. The resultant supernatant was collected as a TX-insoluble fraction.

### Mitochondria isolation

10 to 12 × 10^6^ U2OS cells were trypsinized and collected by centrifugation. The cell pellet was resuspended in 1.1 ml of hypotonic buffer containing 10 mM NaCl, 1.5 mM MgCl2 and 10 mM Tris (pH7.5) and left on ice for 15 min. Swollen cells were next homogenized by Dounce homogenizer with 40 strokes of the tight pestle. Broken cells were then added with 0.8 ml of 2.5x homogenization buffer containing 525 mM D-mannitol, 175 mM sucrose, 2.5 mM EDTA and 12.5 mM Tris (pH7.5) and were centrifuged at 1300*g* for 5 min twice. The resulting supernatant was then centrifuged at 12,000*g* for 15 min. The final supernatant and pellet were regarded as a cytosolic fraction and mitochondria-enriched fraction, respectively. Alternatively, a mitochondria isolation kit for mammalian cells (Thermo Scientific) was also used to purify the mitochondria fraction by following the manufacturer's instructions.

### Oxygen consumption rate analysis

Oxygen consumption rate (OCR) analysis was performed using an XF24 Extracellular Flux Analyzer (Seahorse Bioscience) as described previously (57). Briefly, A549 cells were first plated on culture dishes and transfected with scrambled or specific siRNA for control or depletion or DNA construct for overexpression of mLKB1. 48 h later, cells were replated on XF 96-well microplate (Seahorse Bioscience) at 65,000 cells per well in triplicates. OCR measurement was taken at basal conditions and after adding 1 mM oligomycin, 1 mM FCCP, and 500 nM rotenone/500 nM antimycin mix.

### *In vitro* kinase assay

FLAG-tagged LKB1_L_ and mLKB1 were first transfected into Cos7 cells. 24 h post-transfection, these cells were lysed in lysis buffer containing 25 mM Hepes (pH7.3), 0.15 M NaCl, 1.5 mM MgCl_2_, 0.2 mM EDTA, 20 mM β-glycerol phosphate, 1 mM sodium orthovanadate, 0.3% Triton X-100, 5% glycerol and supplemented with protease and phosphatase inhibitor cocktail. After a 10 min incubation on ice, the samples were given a bath sonication (a single 30 s pulse at high power). After clarification by centrifugation, the cleared lysates were subjected to immunoprecipitation using FLAG antibody-conjugated agarose beads overnight. After three extensive washes, the immunoprecipitated protein kinases were subjected to *in vitro* kinase reaction by incubating with recombinant GST-AMPK fusion protein (activation loop) or Histone H1 protein in a buffer containing 25 mM Tris (pH8.1), 25 mM NaCl, 5 mM MgCl_2_, 200 mM ATP and 0.025% Triton X-100 for 2 h. Reactions were stopped by heating at 95 °C for 10 min.

### Data analysis

Data are represented as Mean ± SD of results of three independent experiments (unless otherwise indicated). Statistical significance between samples was assessed by Student's *t* test.

## Data availability

All data supporting this article are included within the main text and supporting information.

## Supporting information

This article contains supporting information.

## Conflict of interest

The authors declare that they have no conflict of interest with the contents of this article.
