# Supplementary material for: Identification of a novel mitochondria-localized LKB1 variant required for the regulation of the oxidative stress response
Source: J Biol Chem. 2023 Jun 9;299(7):104906. doi: 10.1016/j.jbc.2023.104906 (PMC10404683; doi:10.1016/j.jbc.2023.104906)
Supplement: Supporting Figures S1–S4 [file mmc1.pdf]

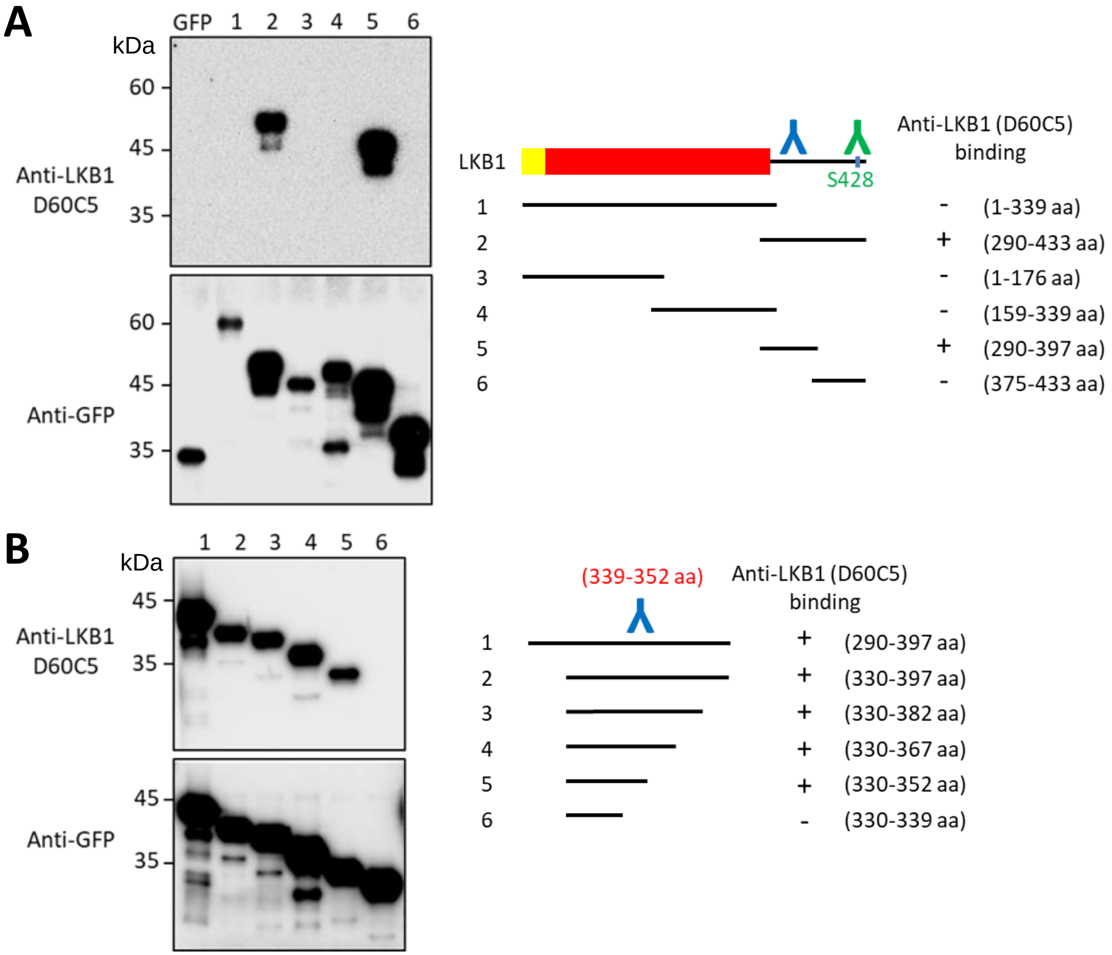

Figure S1. Mapping of antigen binding site of anti-LKB1 (D60C5). (A) Various LKB1 truncated constructs fused with a GFP tag expressed in Cos7 cells were subjected to SDS-PAGE and probed with anti-LKB1 (D60C5) or anti-GFP antibody as indicated. (B) Fine mapping of binding site of anti-LKB1 (D60C5) with smaller LKB1 fragments as indicated.

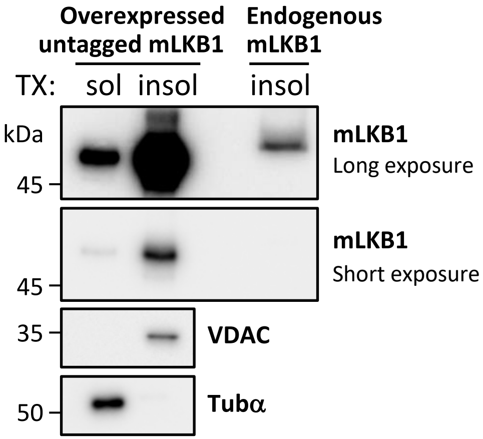

Figure S2. Examination of overexpressed mLKB1 by comparison with endogenous mLKB1. TX-soluble and –insoluble fractions of A549 cells overexpressing untagged version of mLKB1 were resolved on SDS-PAGE and compared to the endogenous mLKB1 by western blot analysis using anti-LKB1 (D60C5). Anti-Tubulin-α and anti-VDAC were used to mark TX-soluble and –insoluble fractions, respectively.

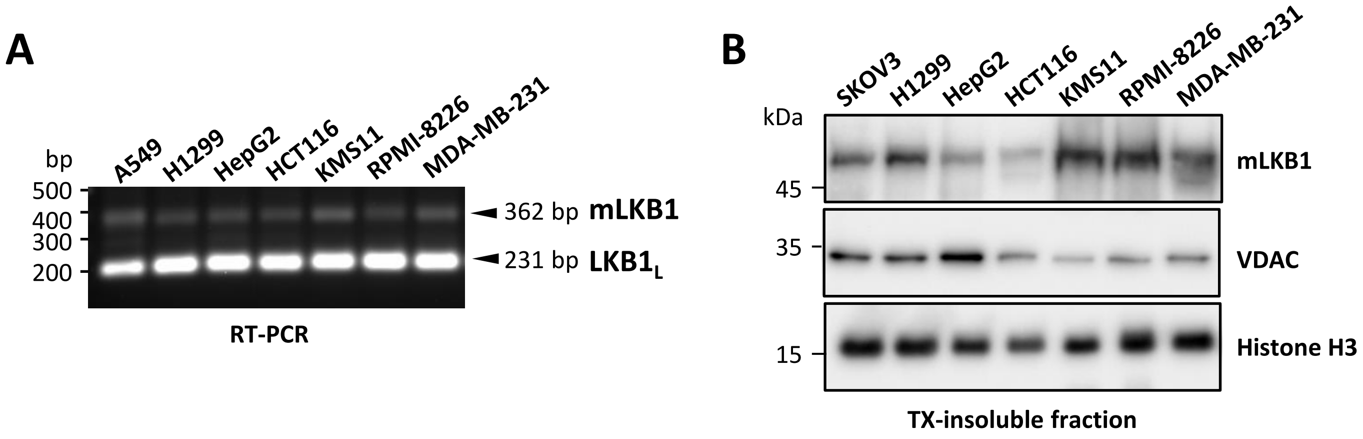

Figure S3. Examination of the expression of endogenous mLKB1 in multiple human cell lines of different tissue origins. (A) Total RNAs were prepared from the indicated cell lines and followed by RT-PCR using primer pair F1/R1 to assess the splicing of LKB1 exon 1b (Fig. 1E). (B) Lysates of indicated cell lines were resolved on SDS-PAGE and probed with anti-LKB1 (D60C5). Anti-VDAC and anti-Histone H3 were used as markers for insoluble fraction and loading control.

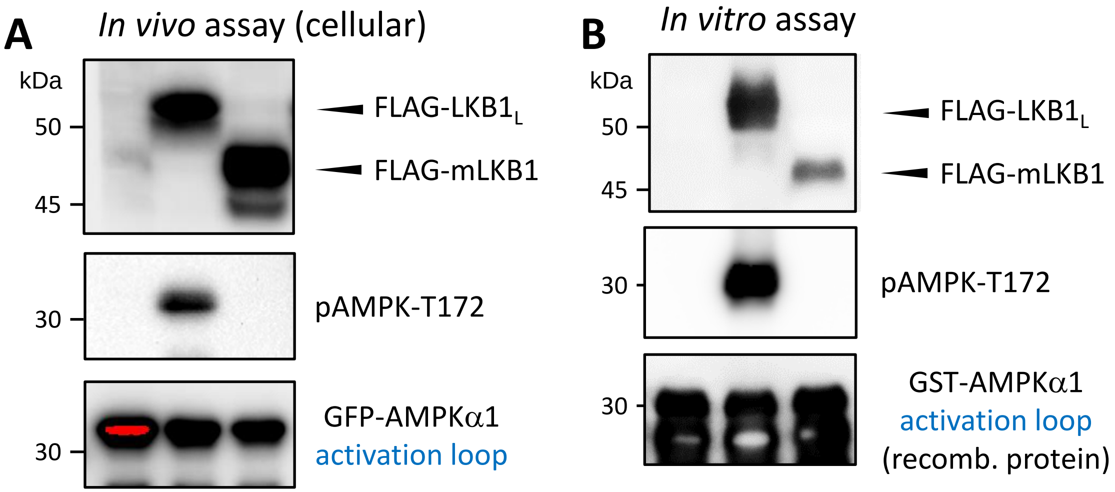

Figure S4. AMPK is not phosphorylated by mLKB1. (A) Lysates of Cos7 cells co-expressing GFP-AMPK (activation loop) with FLAG-tagged $LKB1_L$ or mLKB1 were resolved on SDS-PAGE and probed with antibodies against pAMPK Thr172, GFP and FLAG to assess the phosphorylation level of Thr172 of AMPK. (B) FLAG-tagged $LKB1_L$ and mLKB1 were separately immunoprecipitated from overexpressing cells and tested for their ability to phosphorylate recombinant AMPK (activation loop) in *in vitro* kinase assay.
